# Development of an intervention delivered by mobile phone aimed at decreasing unintended pregnancy among young people in three lower middle income countries

**DOI:** 10.1186/s12889-018-5477-7

**Published:** 2018-05-02

**Authors:** Ona L McCarthy, Ola Wazwaz, Veronica Osorio Calderon, Iman Jado, Salokhiddin Saibov, Amina Stavridis, Jhonny López Gallardo, Ravshan Tokhirov, Samia Adada, Silvia Huaynoca, Shelly Makleff, Marieka Vandewiele, Sarah Standaert, Caroline Free

**Affiliations:** 1 Department of Population Health, Faculty of Epidemiology and Population Health, London School of Hygiene & Tropical Medicine, Keppel Street, WC1E 7HT, London, UK; 2 Palestinian Family Planning & Protection Association, Industrial Zone Wadi Al-Joze, Jerusalem, Palestine; 3 Centro de Investigación, Educación y Servicios – Salud Sexual Salud Reproductiva, Calle 6 de Obrajes Nro. 614 – Casilla, 9935 La Paz, Bolivia; 4 Tajik Family Planning Association, 10 Rudaki Avenue, TC ‘Sadbarg’, 7th floor, Dushanbe, Tajikistan; 5 International Planned Parenthood Federation, Arab World Regional Office, 2 Place Virgile, Notre Dame, 1082 Tunis, Tunisia; 6 International Planned Parenthood Federation, Western Hemisphere Region, 125 Maiden Lane, 9th Floor, New York, NY 10038 USA; 7 International Planned Parenthood Federation European Network, Rue Royale 146, 1000 Brussels, Belgium

**Keywords:** Intervention development, Intervention mapping, mHealth, Tajikistan, Bolivia, Palestine, Contraception, Family planning

## Abstract

**Background:**

Unintended pregnancies can result in poorer health outcomes for women, children and families. Young people in low and middle income countries are at particular risk of unintended pregnancies and could benefit from innovative contraceptive interventions. There is growing evidence that interventions delivered by mobile phone can be effective in improving a range of health behaviours. This paper describes the development of a contraceptive behavioural intervention delivered by mobile phone for young people in Tajikistan, Bolivia and Palestine, where unmet need for contraception is high among this group.

**Methods:**

Guided by Intervention Mapping, the following steps contributed to the development of the interventions: (1) needs assessment; (2) specifying behavioural change to result from the intervention; (3) selecting behaviour change methods to include in the intervention; (4) producing and refining the intervention content.

**Results:**

The results of the needs assessment produced similar interventions across the countries. The interventions consist of short daily messages delivered over 4 months (delivered by text messaging in Palestine and mobile phone application instant messages in Bolivia and Tajikistan). The messages provide information about contraception, target attitudes that are barriers to contraceptive uptake and support young people in feeling that they can influence their reproductive health. The interventions each contain the same ten behaviour change methods, adapted for delivery by mobile phone.

**Conclusions:**

The development resulted in a well-specified, theory-based intervention, tailored to each country. It is feasible to develop an intervention delivered by mobile phone for young people in resource-limited settings.

**Electronic supplementary material:**

The online version of this article (10.1186/s12889-018-5477-7) contains supplementary material, which is available to authorized users.

## Background

In developing regions in 2017, an estimated 89 million pregnancies were unintended, that is, were pregnancies that occurred too soon or were not wanted at all [1]. Unintended pregnancy is associated with a range of negative health and social consequences, for example, poorer access to antenatal care, increased risk of low birth weight and pre-term birth, delays in women’s educational and career achievements and unsafe abortion [2–20]. Women aged 15–24 in low and middle income countries (LMIC) are at particular risk and are more likely to have an unmet need for contraception compared to older women [21]. Women with an unmet need for modern contraception are those who want to avoid a pregnancy but currently use no method or use a traditional method [22]. It is estimated that meeting adolescents’ unmet need for modern contraception would reduce unintended pregnancies by 6 million each year [22].

Tajikistan, Bolivia and Palestine are three LMIC where women are at high risk of unintended pregnancy. In Tajikistan, women have 0.5 more children than desired, with the total wanted fertility rate at 3.3 births per woman compared to the actual of 3.8 [23]. Unmet need for contraception among married 15–24-year-old women is estimated to be 26%, with unmet need for birth spacing the highest among women in this age group compared to women in other age groups [23]. In Bolivia, family planning progress has lagged behind other Latin American countries [24]. In 2008, unmet need for contraception among women aged 15–19, was estimated to be 38% [25, 26]. Among unmarried, sexually active women aged 15–19, 51.9% are not using a method of contraception. Among these women, 84.8% reported not wanting a pregnancy in the next 2 years, yet only 48.8% of them reported using any method of contraception. In Palestine in 2006, an estimated 38% of pregnancies were unintended [27, 28]. In 2014, unmet need for contraception was highest among women aged 20–24, at 15% [29]. While the adolescent fertility rate had decreased substantially in Palestine over the past 20 years, the current adolescent fertility rate of 48 per 1000 women aged 15–19 remains higher than most other countries in the region [29, 30].

The non-permanent ‘effective’ contraceptive methods have less than 10% typical use failure rate at 12 months, i.e. oral contraceptives, injectables, intra-uterine device (IUDs), implants, the patch and the ring (IUDs and implants being the most effective) [31–33]. While effective methods are available in Tajikistan, Bolivia and Palestine, there remain barriers to use. In Tajikistan, oppositional attitudes towards contraception is the most common reason women with a Demographic and Health Surveys-defined (DHS) unmet need [34] provide for not using contraception; 36% of women with an unmet need cite their own opposition and 13% cite their partner’s opposition as the reason for not using contraception [21]. The next most common reasons are infrequent/no sex (28%) and side effects/health risks/inconvenience (15%) [21]. In Bolivia, the main reasons unmarried, sexually active adolescent women who report not wanting a child in the next 2 years provide for not using a method are infrequent sex (54.7%) or not married (51.5%) [25, 26]. Among married women in Palestine not using contraception and not reporting wanting to have a child, the main reasons given for not using contraception were fear of side effects, inconvenience of methods and their husband’s opposition [35, 36].

Mobile phones are now a popular and widely established vehicle to deliver health interventions. There is some evidence from trials that mobile phone-based interventions can improve knowledge about contraception [37, 38] and contraceptive-related behaviours [39–44]. However, all but two of these trials [38, 42] were conducted in the United States and none had low risk of bias [45] according to the Cochrane Collaboration’s tool for assessing risk of bias in randomised controlled trials [46].

In January 2015, the London School of Hygiene and Tropical Medicine (LSHTM) started a collaboration with the International Planned Parenthood Federation’s (IPPF) Member Associations in Tajikistan, Bolivia and Palestine to develop and evaluate an intervention delivered by mobile phone to enhance contraceptive choice among young people in each country. At this development stage of the project, we included both young women and men because women in these settings have reported that their male partners’ attitudes influence their use of contraception. To the best of our knowledge, this research is the first to develop such an intervention for young people in these countries. This project helps fill the research gap regarding the development of mobile phone interventions for contraception in LMIC.

## Methods

### Intervention development approach

Intervention Mapping (IM) guided the development of the interventions [47, 48]. IM is a protocol for the systematic development of health behaviour change interventions. It is a cumulative process that often necessitates moving back and forth through the following steps: (1) needs assessment; (2) specifying behavioural change to result from the intervention; (3) designing the intervention components by selecting behaviour change methods; (4) producing and refining the intervention content; (5) planning intervention implementation and (6) planning intervention evaluation. This paper describes steps 1–4 and compares the results across the countries.

### Needs assessment

The needs assessment aimed to understand unintended pregnancy and contraceptive use in each context. Activities included 1) establishing a project planning group 2) a literature search 3) focus group discussions (FGDs) and interviews with the target group and 4) interviews with local service providers.

Each country’s project planning group was a collaboration between the local partner, the research partner and the three IPPF Regional Offices. The local partners consisted of the Executive Director, Research Assistants, and various other employees of the organisation that contributed to the development process in different capacities. The research partner designed and managed the research. Staff from Regional Offices attended meetings and facilitated communication about the research between the research partner and the local partner.

Relevant articles were identified through the research partner’s existing knowledge, recommendations by the local partners, a Google search for grey literature and a search of MEDLINE. The results of the literature search informed the discussion guide used in the FGDs and interviews. Remote meetings were held from February to September 2015 to plan and organise the field research, which took place in July 2015 in Tajikistan, August/September 2015 in Bolivia and October 2015 in the West Bank, Palestine.

The FGDs and interviews with the target group explored their knowledge of and attitudes toward contraceptive methods, perceived barriers in using and confidence in communicating about them (see Additional file 1 for the target group discussion guide). This information was used to better understand the personal, socio-cultural and socio-economic factors involved in contraceptive use in each setting. The consultations also aimed to understand how amenable young people are to trying new contraceptive methods, their patterns of mobile phone use, preferences for intervention content and views on privacy regarding receiving contraceptive information on their mobile phone. The interviews with providers explored similar topics from a provider perspective (see Additional file 2 for the provider discussion guide). The research partner trained local research staff in FGD and interview facilitation and research ethics. The number of groups and interviews estimated (up to ten of each) was based on previous intervention development experience [49, 50].

Target group participants were identified by convenience sampling through the local partners’ youth volunteer network and services in Dushanbe and Vahdat (Tajikistan), El Alto (Bolivia) and Ramallah, East Jerusalem, Hebron and Bethlehem (Palestine). Women and men were eligible if they were legally able to give independent informed consent (age 14 in Tajikistan, 18 in Bolivia and 18 in Palestine). There was no upper age limit but each local partner focused on recruiting younger participants as this most closely matched the target group ‘young people’ [51, 52]. Providers were affiliated with each local partner.

Each FGD and interview was conducted by a research staff member who was a native speaker of the local language (and in most cases also spoke English) and was attended by a bilingual (English and local language) research staff member who took detailed notes. Immediately after, the facilitator/interviewer relayed the information to the research partner who made detailed notes in English. The FGDs were comprised of participants of the same gender and facilitated by a staff member of the matching gender. The FGDs and interviews were held at the service or at a location hired specifically for this purpose. The FGDs and interviews were audio recorded. The FGDs lasted up to 90 min and the interviews lasted up to 60 min. Resources allowed only for the FGDs in Bolivia to be transcribed and translated into English. We conducted a descriptive thematic analysis of the FGDs and interviews by examining the discussion notes related to each theme in the discussion guide. An information technology partner consultant based in the United Kingdom reviewed the local mobile phone operators and identified local technology partners.

We depicted the results of the needs assessment visually in a ‘logic model of the problem’.

### Specifying behavioural change

The needs assessment led to the specification of the desired behaviours for target group to accomplish as a result of the intervention (behavioural outcomes) and of the desired changes in the environment to occur as a result of the intervention (environmental outcomes). The performance objectives for the behavioural outcomes were then specified by identifying the smaller actions that are logically required to perform the outcome. The determinants of these actions were specified from the literature search and insights from the FGDs and interviews and behaviour-oriented theories [47]. Mapping these against one another in a matrix enabled the identification of the most immediate behaviours that the intervention aims to alter in the individual (change objectives). While the environmental outcomes were specified, it was beyond the scope of the project to develop an intervention to target these conditions therefore the performance objectives and determinants for the environmental outcomes were not specified.

### Designing the intervention

This step involved choosing theory-informed behaviour change methods to include in the intervention and deciding how to deliver them [47, 48, 53]. Potential methods were identified by considering: 1) authors’ report of the methods used in existing effective interventions for contraception [43, 54, 55] and 2) the methods shown to modify each determinant according to the IM taxonomy [48]. (The IM taxonomy describes the behaviour change methods that have been shown to modify different types of behavioural determinants.) Throughout the process, the conditions under which the methods can be effective were considered (the ‘parameters for effectiveness’) [47, 48].

### Producing and refining the intervention content

The intervention content was written when behavioural change was specified and the methods and theoretical basis of the intervention were identified. The research partner wrote the initial content. It was then reviewed by the local partner for cultural appropriateness and amended with the research partner. Next, the target group was consulted for their views on the tone, acceptability and comprehensibility of the content. The content was refined with the target group after each consultation and tested until it was acceptable to them.

## Results

### Needs assessment

The factors reported in the published literature that influence contraceptive use and reasons for unmet need in LMIC and in Tajikistan, Bolivia and Palestine are summarised in the Background.

### Focus group discussions and interviews with the target group

Eight FGDs each were conducted in Tajikistan and Bolivia and five were conducted in Palestine; one user interview was conducted in Tajikistan, two in Bolivia and four were conducted in Palestine (see FGD and interview demographics in Table 1). In Tajikistan and Bolivia, we stopped the FGDs and interviews when no new data was emerging in relation to the themes in the discussion guides. The FGDs and interviews coincided with the escalation in conflict in the West Bank in the first few weeks of October 2015. Due to logistical challenges related to this, we were unable to conduct more than five FGDs in Palestine.

**Table 1:** Focus group discussion and interview demographics

	Tajikistan *n* = 78 n (%)	Bolivia *n* = 64 n (%)	Palestine *n* = 35 n (%)
Number of participants			
FGD1	10	5	10
FGD2	8	8	Not attended
FGD3	10	10	Not attended
FGD4	8	5	4
FGD5	15	10	3
FGD6	9	10	7
FGD7	9	7	7
FGD8	8	7	Not attended
Interviews	1	2	4
Age			
15–19	37 (47.4)	26 (40.6)	2 (5.7)
20–24	37 (47.4)	36 (56.3)	26 (74.3)
25–30	4 (5.1)	2 (3.1)	5 (14.3)
Missing	0	0	2 (5.7)
Gender			
Male	33 (42.3)	28 (43.8)	13 (37.1)
Female	45 (57.7)	36 (56.3)	22 (62.9)
Missing	0	0	0
Residential area			
City	50 (64.1)	not collected^b^	10 (28.6)
Other^a^	28 (35.9)		24 (68.6)
Missing	0		1 (2.9)
Occupation			
Working	20 (25.6)	4 (6.3)	5 (14.3)
Unemployed	8 (10.3)	0	3 (8.6)
Full-time parent	1 (1.3)	0	3 (8.6)
In education or training	49 (62.8)	60 (93.8)	19 (54.3)
Missing	0	0	5 (14.3)
Pregnancy intention (current)			
Avoid	11 (14.1)	38 (59.4)	13 (37.1)
Unsure/not avoid/do not mind	29 (37.2)	14 (21.9)	18 (5.1)
Not sexually active	30 (38.5)	12 (18.8)	2 (5.7)
Missing	8 (10.3)	0	2 (5.7)
Current method			
None^c^	51 (65.4)	20 (31.3)	13 (37.1)
Condoms only	22 (28.2)	31 (48.4)	3 (8.6)
Withdrawal only	0	1 (1.6)	3 (8.6)
Condoms and withdrawal	2 (2.6)	0	0
Calendar-based only	0	5 (7.8)	2 (5.7)
Effective method^d^	3 (3.8)	5 (7.8)	8 (22.9)
Condoms and calendar-based	0	1 (1.6)	1 (2.9)
Lactational amenorrhea method only	0	0	1 (2.9)
Condoms and lactational amenorrhea method	0	0	1 (2.9)
Missing	0	1 (1.6)	3 (8.6)

^a^’Other’ in Tajikistan is Vahdat, a large town 10 km outside of the capital Dushanbe; ‘Other’ in Palestine is village or refugee camp

^b^Participants from El Alto, La Paz or close surrounding areas

^c^Includes participants not sexually active

^d^Oral contraceptives, injectables, intra-uterine devices (IUDs), implants, the patch or the ring

### Use of mobile phones

Use of mobile phones was nearly ubiquitous in all three countries but there was some variation in terms of the types of phones used and mobile Internet access. Most participants owned a smart phone, and if not, they owned a feature phone. Around five female participants in Vahdat, Tajikistan said that they did not have a mobile phone at all. In Tajikistan and Bolivia, it was more common to have regular mobile Internet access than in Palestine. Of those who owned a smart phone, Android phones were the most popular, with participants in Bolivia saying iPhones were for people of “high status”. Participants in Tajikistan and Bolivia accessed the Internet through their phones. A few participants in Tajikistan said that access was sometimes restricted due to insufficient funds to support Internet connectivity. In the rural areas of Tajikistan, mobile Internet connection is expensive and electricity is restricted to three to 5 hours a day in the winter, restricting the ability to charge the battery. In Bolivia, participants said that they do not have Internet access on their phones all the time and buy the smallest data package possible to support use of Facebook and WhatsApp. In Palestine, many did not have regular Internet access on their phones and if they did, most access the Internet by Wi-Fi only; those who sometimes access the Internet though their mobile data said that it is common for the connection to be lost.

### Contraception support

Participants in all three countries were very enthusiastic about receiving information about contraception on their phone. Participants in Tajikistan thought that acquiring accurate information would improve young people’s attitudes towards contraception. In Bolivia, participants expressed very strongly the need for more information and talked about the convenience of being able to look at their phones for contraceptive information without having to ask anyone. In Palestine, while most young people wanted contraceptive information delivered on their mobile phones, female participants were more supportive of the idea than the male participants. A group of male participants said that they may read the information and benefit from it but it would be considered a joke and not taken seriously. A male participant said that contraceptive information delivered to phones is new in Palestinian society and that it is important that the information be given in a respectful way as “people feel shame about these issues”.

### Intervention delivery preferences and privacy

Participants expressed a range of mobile phone media ideas for intervention delivery, such as videos, pictures and animations. In general, participants preferred to receive contraception support through short message. In Tajikistan, participants thought it was helpful to save the messages to read later at a convenient time and because messages are easy to delete if they want to prevent others seeing them. However, a few were less comfortable receiving support by short message because they were perceived as less private. Bolivian participants, while interested in a variety of intervention delivery modes, preferred information to be sent by simple instant messaging through an app or text messaging. Palestinian participants preferred text messages for intervention delivery, with some saying that they wanted messages delivered by app instant messaging.

Most participants reported that they do not share their phones. Participants in Tajikistan said that if they do share their phone, they lend their phone to friends or family to take photos, play games, listen to music and browse social networks. One female participant in Palestine said that sometimes she asks her children to check her phone when she is busy and she was concerned that they would see the messages. Another Palestinian participant said that she would share her messages with her husband to “educate him”. A few participants mentioned concerns about others seeing the messages and having information about contraception on their phone but the majority were not concerned if they can password protect the phone.

### Intervention content

Preferences for intervention content were similar across the countries, with some context-specific preferences. In all countries, participants wanted to hear about other people’s experiences, particularly “success stories”, with using contraception. In Bolivia, participants thought that hearing real stories or “testimonies” from people who have had experience using different contraceptive methods would make them feel confident in trying new methods. Some participants in Tajikistan however, did not want information in the form of stories because they value the advice from a specialist over advice from a peer. In Palestine, participants said that they would trust “scientific” information. Participants in all countries wanted clear and concise information about the advantages and disadvantages of the different methods, how to use them and where they can obtain them. Tajik participants said that the content should not contain difficult terminology and the ‘voice’ should not be young, as this would be perceived as less trustworthy. A female group in Bolivia said that they wanted “little messages” giving advice and not telling them what to do. In terms of frequency, Tajik participants thought that one to two brief messages a day is acceptable, Bolivian participants wanted 1–3 messages a day and Palestinian participants said that they wanted around three messages per day.

### Knowledge about contraception

Across all of the countries, there was a good level of awareness of the effective methods, but a lack of comprehensive knowledge such as the efficacy, advantages and disadvantages of the methods, how to use them and how they work. Participants agreed that young people do not have adequate information about contraception and were curious to know more about the range of methods available.

### Attitudes and beliefs towards contraception

Across the countries, participants expressed a range of negative beliefs about effective contraception. Common beliefs were that hormonal contraception (including the non-hormonal IUD) is damaging to the health of the women, not effective in preventing pregnancy, causes heavy and irregular bleeding (IUD), infertility and weight changes. In Bolivia, participants mentioned that the IUD can rust in the uterus and causes cancer and that hormonal contraception makes people “stop being normal”. Tajik participants mentioned that the IUD can grow into the skin. A few female participants in Palestine spoke favourably of the IUD but most thought that the metal in the IUD is harmful and causes an irregular menstrual cycle. A female group in Palestine though that the pill causes anxiety and nervousness.

In Tajikistan and Palestine, most participants approved of the concept of family planning and thought that society did as well. This was mainly because they believed that it helps families plan and assess their economic situation. Palestinian participants thought that it is better to use “natural” methods in the first year of marriage, with a male group saying that using contraception in early marriage will create problems.

### Communication about contraception

In Tajikistan and Bolivia, participants expressed a lack of confidence communicating about contraception with partners, parents and providers. Participants in Palestine thought that it was possible to talk to close friends and mothers about contraception. A female Palestinian participant said that it is common for partners to talk about sex before marriage (it was not clear if this was discussion about contraception or just sex). In Tajikistan, a group of male participants said that it can be difficult to talk about contraception with a partner that they have been in a relationship with for a while because they fear that their partner would take offense. A Tajik female group thought that it is difficult to negotiate contraceptive use if they did not want a pregnancy at the time and their partner did. Bolivian participants said that they are confident talking to close friends (friends of opposite gender must be very close) but not confident talking to partners unless they know them very well and trust them. They said that talking about contraception usually happens after they begin a sexual relationship and if a discussion about contraception were initiated too early with a partner, the partner would judge them as promiscuous.

In all countries, participants were not confident talking to providers about contraception because of the cultural stigma surrounding sex before marriage, concerns about confidentiality and fear of being judged. In Bolivia, many participants spoke of negative experiences at services.

### Environmental factors

In Tajikistan and Palestine, participants expressed that the strong stigma surrounding sex before marriage in their cultures creates concerns about confidentiality and prevents them from accessing services. In Bolivia, this stigma was implicit, with participants talking about fear of being judged for seeking sexual and reproductive health information and services. Many participants in Bolivia spoke of experiences of being given “bad looks” for attending services (pharmacies and reproductive health services) and fear of feeling embarrassed and ashamed. There was a very strong fear in Tajikistan and Bolivia that others (people in the community) will know that they attended, judge them and spread “rumours”. There was general agreement across the discussions in Palestine that unmarried people accessing services is not accepted and is highly stigmatised. A female group in Palestine felt that they would be judged by providers and they will be asked if they are married or not if they attend a service. They also felt that they would be judged by their community if they went to a service and were not married.

In Palestine and Tajikistan, participants spoke of pressure to begin childbearing soon after marriage (Tajik participants mentioned 9 months after). If they do not, they fear that they will be judged and considered unhealthy. Many participants in Palestine talked about the societal pressure from their mother-in-law, friends and neighbours to conceive soon after marriage. If a married woman in Palestine prefers to complete her university degree, participants said that she is pressured to conceive and if she does not, the community assumes that she has fertility problems. A group of female participants in Palestine mentioned that it can be difficult to reach the service because of checkpoints.

Partners and the educational system are environmental agents that also contribute to the problem. This is apparent from participants’ report of the lack of partner communication about contraception before sexual activity and their lack of comprehensive knowledge about contraception.

### Interviews with service providers

Six interviews with service providers were conducted in Tajikistan (5 doctors and 1 nurse outreach worker) and five were conducted in Bolivia (1 health advisor, 1 educator, 1 nurse and 2 doctors). Due to logistical challenges as a result of the escalation in conflict in the West Bank, only one provider interview was conducted in Palestine.

Providers in all countries said that young people are generally aware of the range of methods. However, many said that they do not know how they work or how to use them and want more information on these topics. In Bolivia, lack of information and misinformation is what most providers viewed as the greatest barrier for young people using new methods, along with the cost of contraception.

Providers in Tajikistan and Bolivia thought that young people are eager for more information about contraception and that providing this would help more young people use methods. Bolivian providers also said that more young people would use methods if they were affordable. The Palestinian provider said that in the pre-marital counselling sessions that she conducts, young people ask questions that make it obvious that they are already sexually active. Her perception is that young people want more information because many are having sex before marriage. She thought that providing young people with other people’s success stories would help young people try new methods.

Providers in Tajikistan thought that attitudes towards reproductive health are changing and that people are thinking about the consequences of sexual activity before engaging in it. One provider said that young people have a positive attitude towards contraception. Providers said there is a lot of misinformation among young people in Bolivia and that they fear the perceived side effects, such as the infertility they associate with oral contraceptives. The Palestinian provider said that that young people think that the IUD makes them nervous and infertile.

Tajik providers commented on how religion does not accept sexual activity before marriage and that the mother-in-law has a great amount of influence on her daughter-in-law’s contraceptive use. Providers said that young people (particularly young unmarried people) do not even try to attend services covertly because they are too concerned that someone will find out. A provider in Bolivia said that barriers stem from problems with the family and that these women are more at risk for early sexual debut. Some providers in Bolivia also mentioned machismo as a barrier in that if a woman is using contraception, men will perceive her as “horny”, which is threatening to their masculinity.

**Fig. 1:**
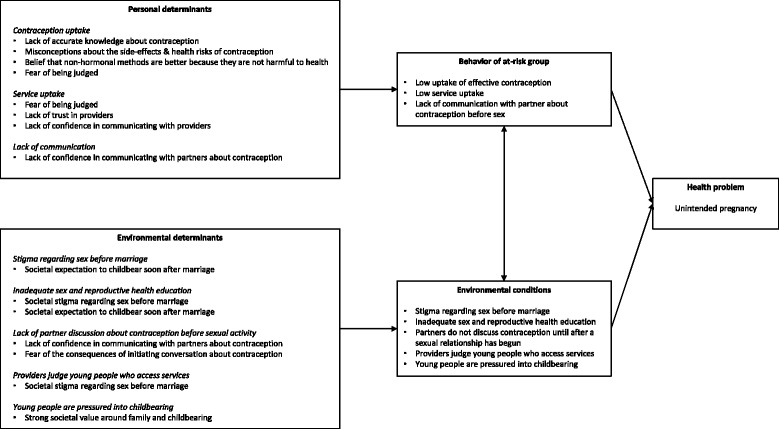
Logic model of the problem

See Fig. 1 for the logic model of the problem.

### Specifying behavioural change: Results

#### Behavioural outcomes

Based on the needs assessment results, we specified the key behavioural and environmental outcomes (Table 2).

**Table 2:** Behavioural and environmental outcomes

Behavioural				
1. Young people use effective contraception	2. Young people access reproductive health services		3. Young people communicate with partners about contraception before sexual activity	
Environmental^a^				
1. Sex before marriage is less stigmatised	2. Young people have access to comprehensive and accurate sexual and reproductive health education	3. Partners discuss contraception before a sexual relationship has begun	4. Providers do not judge young people who access services	5. Young people are not pressured into childbearing

^a^Not targeted by the mobile phone intervention

### Theory, performance objectives and determinants

**Fig. 2:**
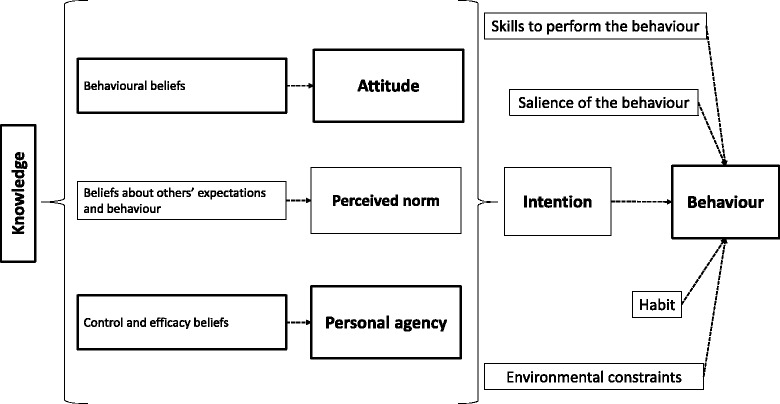
This project's Integrated Behavioural Model

The Integrated Behavioural Model (IBM) [56] is the overarching framework for the intervention. This project’s IBM was adapted to include knowledge as a fundamental determinant of behaviour, as in the Information-Motivation-Behavioural Skills Model [57] (Fig. 2).

Table 3 presents the behavioural outcomes, performance objectives and determinants. The literature supports the determinants knowledge [22, 58–67] and attitudes [22, 58, 60–64, 68–72], as important influences contraceptive use. It was clear from the needs assessment that accurate information about effective contraception was low. Providing accurate information in a context where there is none or very little, may change people’s beliefs. Attitudes and intention were verified by behaviour-oriented theory [56, 73, 74]. Personal agency [75] in the IBM is comprised of efficacy [56, 76, 77] and control beliefs [56, 78]. The addition of perceived control acknowledges that while a member of the target group may feel confident in using a contraceptive method, they may not feel that they have control over whether they use it.

**Table 3:** Behavioural outcomes, performance objectives and determinants

Behavioural outcomes	Performance objectives	Determinants
Use effective contraception	po1.1 Choose a method	*Knowledge* about the effective methods
		*Attitude* towards using effective methods
		*Intention* to use effective methods
		*Personal agency* in choosing an effective method
	po1.2 Acquire the method	*Knowledge* of where to get effective contraception
		*Attitudes* about acquiring effective contraception
		*Intention* to acquire effective contraception
	po1.3 Use the method correctly	*Knowledge* about how to use effective contraception correctly
		*Intention* to use effective contraception correctly
		*Personal agency* in using effective contraception correctly
Access reproductive health services	po2.1 Locate a service	*Knowledge* of where to get effective contraception
		*Attitudes* about acquiring effective contraception
		*Intention* to locate a service
	po2.2 Travel to the service	*Intention* to travel to a service
		*Personal agency* in traveling to a service
	po2.3 Communicate effectively with providers	*Personal agency* in communicating with providers
Communicate with partners about contraception before sexual activity	po3.1 Initiate conversation with partner about contraception	*Attitudes* towards partner’s approval of contraception
		*Intention* to initiate conversation about contraception
		*Personal agency* in initiating a conversation about contraception
	po3.2 Clearly state own preferences regarding contraception to partner	*Intention* to clearly state contraceptive preferences to partner
		*Personal agency* in clearly stating contraceptive preferences to partner
	po3.3 Listen to partner’s preferences regarding contraception	*Intention* to listen to partner’s contraceptive preferences
		*Personal agency* in listening to partner’s contraceptive preferences

While intention was identified as a determinant, the intervention does not influence it directly, rather, the intervention aims to influence the behavioural (attitudinal), efficacy and control beliefs identified in the needs assessment, which all influence intention.

### Change objectives

Crossing the behavioural outcomes, performance objectives and determinants, it was possible to specify the most immediate behaviours that the intervention aims to alter (see Table 4 for a partial matrix and Additional file 3 for the complete matrix). The change objectives for all countries were the same, except for a2.1.3, which only applies to Bolivia, because this was a specific cultural norm that emerged from the needs assessment (Additional file 3).

**Table 4:** Partial matrix of change objectives

	Determinants			
Performance objectives				
	Knowledge	Attitudes	Intention	Personal agency
*Young people will…*	*Behavioural outcome 1: Use effective contraception*			
*po1.1 Choose a method*	k1.1.1 Name the effective methods k1.1.2 Describe how the effective methods work k1.1.3 List the risks & benefits of the range of effective methods	a1.1.1 Express positive attitudes towards the effective methods a1.1.2 Recognise that hormonal methods are not less healthy than non-hormonal methods a1.1.3 Differentiate between real potential side-effects and misconceptions a1.1.4 Recognise that an experience of side-effects in one method may not occur in another method	i1.1.1 Assess options i1.1.2 Express intention to choose effective contraception	pa1.1.0 Express personal agency in choosing an effective method despite fears of being judged by society (married or not married)

### Designing the intervention components: Results

#### Practical application of the methods

After the needs assessment, through frequent discussions, the project planning groups decided that the intervention would be delivered through short, one-way messages.

#### Behaviour change methods

Descriptions of the methods used in the effective contraceptive interventions, were limited [39–42, 79–82]. Two of these trials [83, 84] reported using *motivational interviewing*, which could not be successfully delivered though automated messages.

Using the IM taxonomy, we identified methods previously shown to be effective in modifying the determinants (knowledge, attitudes, personal agency) and considered their use for the intervention. Because the methods had to be delivered through short instant message, they required adaptation and there were only two methods from the initial list whose parameters for effectiveness (the conditions under which the methods can be effective) could be fully satisfied (*belief selection* and *tailoring*). While the parameters for most methods could not be fully satisfied, they were used if they could partially or potentially be satisfied. The final methods included in the intervention are [48]: *belief selection, facilitation, anticipated regret, guided practice, verbal persuasion, tailoring, cultural similarity, arguments, shifting perspective* and *goal setting* (see Additional file 4 for details regarding the behaviour change methods).

### Producing and refining the intervention content: Results

The initial message sets were largely similar across the countries given the similar results of the needs assessment. Feedback from the Tajik partner on the initial set of messages was regarding specifics about the methods available in the country. Feedback from the Bolivian partner was regarding the meaning of some of the messages, which was clarified through discussion. The Palestinian partner’s initial feedback was them seeking clarification for the rationale behind some of the messages and suggesting a few changes to maintain cultural sensitivity.

In Tajikistan, 34 young people (17 female and 17 male, the majority of whom were volunteers at the organisation) tested the intervention over four rounds. The Bolivia intervention was also tested over 4 rounds and involved 47 young people (29 female and 18 male) who were a mix of the organisation’s ‘young leaders’, university students who were non-service users and women who were sexually active and did not report using effective contraception. The intervention in Palestine was tested over three rounds with 17 people (eight female and six male) five of whom were volunteers in the organisation and 12 of whom were non-service users.

In general, feedback across the countries was that the messages were helpful and they were enthusiastic about the intervention. There were no clear differences in acceptability of the messages by gender. The Tajik volunteers wanted more clarification about how the methods work, which appeared to be for reassurance that they were safe to use. They also said that in general, they wanted the messages to be more “interesting and joyful”. Target group feedback in Bolivia was that the messages should be more light-hearted, contain emoji’s within the messages, “curiosities” about contraception and messages about “pop stars”. This was in contrast to Palestine, where the target group preferred messages that were “scientific”. Palestinian volunteers reported that the messages overall were reassuring and socially acceptable. There were some messages that they said sounded too negative and they suggested rewording to sound more reassuring. The research partner incorporated feedback after each round of testing and tested a revised set of messages in the following round.

#### Final intervention

The fundamental structure of the intervention is the same across the countries. Each intervention is designed to target the belief-based constructs identified in the needs assessment (instrumental attitude, self-efficacy and perceived control) in relation to contraception use, access to services and communication with partners about contraception. Each intervention provides accurate information about the effective contraceptive methods available in the country and aims to support young people in believing that they can influence their reproductive health. The messages are mapped to their corresponding change objective/s, behaviour change method and behavioural outcome (however, not all messages address a change objective, contain a behaviour change method or target a behavioural outcome).

The interventions contain the same ten behaviour change methods and similar content, with minor contextual variations resulting from the testing (see Table 5 for a sample of the intervention messages).

**Table 5:** Sample intervention messages

Tajikistan	Bolivia	Palestine
Specialists have tested hormonal contraceptives many times and found them to be safe.	Some people think that hormonal methods are less healthy than non-hormonal methods. Hormonal methods are safe.	Some people think that hormonal methods are less healthy than non-hormonal methods. Hormonal methods are safe under medical supervision.
The most effective methods are: pills, IUD, implant and injection. These methods are over 99% effective if used correctly.	The most effective methods are: pills, t-copper (intrauterine dispositive), implant, injection & patch. If used correctly, less than 1 out of 100 women will get pregnant in a year if they use one of these .	The most effective and available methods in Palestine are: pills, IUD, implant, injection, patch. These methods are 99% effective if used correctly.
Some woman may not have a period when on the injection. Some people say that they like not having periods because they can be painful and inconvenient.	Bleeding may change or even stop with the injection. Some people like not having a period.	The bleeding cycle may change or even stop with the injection. Some people like not having a period.
Making decision about contraception with a partner makes it more likely that you will avoid an unintended pregnancy.	Making decision about contraception with a partner makes it more likely that you will avoid an unintended pregnancy.	Making a decision about family planning with your husband helps you avoid an unintended pregnancy.
Providers see young people with different kinds of lifestyles choose contraception.	Providers see young people, married and not-married, all day and help them choose contraception. They want to help rather than judge.	Providers help people of different lifestyles regarding family planning.
Some young people worry that providers will judge them. Remember, it’s about your health and you can choose what is right for you.	Some young people worry about being judged by other people too. Your health is what’s important. It’s your body and your right .	Remember it’s about your health and you have the right to choose what is right for you regardless of how others think and feel.
Think about your situation and what is right for you. If you decide to use contraception without your partner knowing, the IUD and implant are easy to hide.	If you are worried, there are methods that you can use without others knowing.	If your husband disapproves, talk to him about why you believe that it’s a good decision for you. The IUD and implant are easy to keep private.

The messages are tailored according to marital status (a proxy for sexual activity) and gender in Tajikistan and Palestine (male messages in Bolivia were not developed due to an early decision that the intervention would be evaluated with women only). The message sets start with 6–7 days of messages with information about what they will receive over the next 120 days, how to stop the messages, who to contact if they change their number, how to keep the messages private and information about who to call if they feel unsafe as a result of someone reading the messages. Over the next 112–113 days, intervention recipients receive 0–3 messages a day covering the following: accurate information about the effective methods; short quotes derived from real quotes from the target group regarding their views and experiences using each method; messages targeting specific misconceptions about contraception identified in the needs assessment; messages providing support for communicating with partners about contraception; messages that aim to reassure recipients that it is a provider’s job to maintain confidentiality; information about the cost of the different methods and where to obtain contraception and messages emphasising the importance of method switching rather than discontinuation. On day 119 and 120, the message sets include two messages that indicate that the messages have ended and provide reassurance that the information that they provide is confidential.

## Discussion

### Main results

The application of Intervention Mapping resulted in one intervention, tailored for Tajikistan, Bolivia and Palestine. The interventions are well specified, with each step in the development process documented. The needs assessment revealed that mobile phone ownership is widespread in each country and that young people are eager to receive contraceptive support on their mobile phone. Young people lacked comprehensive knowledge about contraception and expresses a range of negative beliefs about effective methods. They expressed a lack of confidence communicating about contraception and mentioned various environmental barriers to use. This study demonstrates that it is feasible to develop an innovative, comprehensible, acceptable intervention delivered by mobile phone with and for young people in resource-limited settings.

### Strengths and limitations

A strength of the development is the participatory design. Young people were an integral part of the process and strengthened the intervention. Target group participants were a heterogeneous group, particularly in terms of age, gender and residential area. This is the only study we are aware of that has used the same approach to develop, in parallel, interventions delivered by mobile phone in three different contexts. Because the intervention content is mapped to the corresponding change objectives and behaviour change methods, it is well specified.

While there were strengths in conducting this multi-country research, it was also a challenge to spread our resources across the three countries. We conducted a pragmatic study using qualitative methods to explore the key themes related to unintended pregnancy and contraceptive use identified in the literature, to inform the development of the intervention. Working in one country would have allowed greater time and resources to conduct a more in depth qualitative study. It is possible that a more in depth, inductive approach could have produced a slightly different intervention. However, it is reassuring that our findings are in line with the global literature.

The needs assessment revealed that there are powerful environmental influences, such as stigma surrounding young people using contraception and pressure to child bear soon after marriage. While not mentioned explicitly by young people, this cultural stigma is likely a result of religious belief that maintains sexual activity is reserved for marriage (Islam in Tajikistan and Palestine and Christianity in Bolivia). A potential limitation of this project is that the delivery mechanism was pre-specified. Targeting these important environmental conditions would likely require a broader intervention than messages delivered by mobile phone.

Another limitation is that the target group was defined at the start of the project. The needs assessment revealed that unmet need is greatest in all three countries in a slightly older age group, i.e. 19–30. Due to funding restrictions, the focus group discussions in Tajikistan and Palestine were not transcribed and translated into English. The research partner who wrote the first draft of the intervention relied on the report of the trained facilitators and note takers.

In Palestine, the increase in conflict that coincided with the fieldwork meant that some FGDs were not well attended and only one provider interview was conducted. A consequence of this is that the views of younger people may not have been adequately explored because only two participants were aged 15–19. However, the message testing provided reassurance that the intervention was appropriate and acceptable.

In Bolivia and Tajikistan, participants in the FGD were members of the youth network or recruited by the youth network. It may be that this group was more informed than people not connected with services in any way. Still, there were widespread negative beliefs and low levels of comprehensive knowledge about contraception. Another way that this may have hindered the development is that this group may be more likely to find a mobile phone intervention for contraception acceptable because they had greater exposure to the topic and therefore may be more comfortable with it. In addition, young people who have no connection with services may have greater confidentiality concerns.

Most participants were either employed or in education or training. The project could have benefitted from the inclusion of more participants in other occupational categories.

It was only possible for the research partner to train each local team remotely in the testing procedures. The research partner relied on their report of the results, some of which were more detailed than others. It is not clear if this variability was due to the testing facilitators, the target population or both. If the project had more time and resources, we would have tested the intervention with a wider range of people (e.g. more at-risk groups and people who were not connected to the youth networks).

Intervention Mapping provides a comprehensive guide for developing complex health interventions targeting both individuals and environmental agents. This project, however, is smaller in scope and targets individuals only. The determinants were not quantitatively verified [85] and their importance or changeability was not assessed. The adaption of the behaviour change methods for delivery by mobile phone and lack of fully accounting for the parameters for effectiveness is likely to result in some loss of meaning [47]. While there are various modes in which to deliver content via mobile phones, strictly speaking, it is counter to Intervention Mapping to rule out non-mobile phone options from the start. Despite this, a mobile phone intervention emerged as highly acceptable and appropriate mode of intervention delivery.

Although participants in Bolivia mentioned that cost was a barrier to contraceptive use, the intervention could not address this. Even if the intervention is successful in improving attitudes towards the methods, uptake may not be improved if cost remains a barrier.

### Comparisons with existing research

The results of the consultation with the target group align with existing research regarding the attitudinal factors that influence contraceptive use, i.e. that concern about the side effects or health risks are the most commonly expressed beliefs [22, 58, 60–63, 68–72]. Consistent with other research [59, 62, 66, 86, 87], this study confirms similar environmental barriers to contraceptive use, such as stigma regarding sex before marriage and the societal value around family and childbearing. Other research involving young people has shown that participants are willing and eager to receive contraceptive information on their mobile phone [39, 41, 88–90]. While there was variation in mobile Internet access among participants, mobile phone use was nearly ubiquitous. This reflects the global growth of mobile phone subscriptions, which has been slower in LMIC compared to higher income countries but is rising [91].

### Implications

The fact that the beliefs identified in the needs assessment were similar to the beliefs in the literature suggests that the intervention is likely to be somewhat generalizable. The approach that we used to develop the intervention was successful in three culturally different settings, which highlights its broad applicability. Adaptation of the intervention to different settings could be more straightforward than usual, because the intervention is well specified.

## Conclusions

The intervention development process resulted in one intervention, tailored to three contexts. The process exhibited how similar factors contribute to contraceptive use across three geographically and culturally unique LMIC settings. This project contributes to the field of contraception intervention development and mobile health. It has taken forward the practice of adapting behaviour change methods for delivery by mobile phone. This contribution highlights the importance of developing interventions using a systematic approach. The intervention has been evaluated by randomised controlled trial among men and women aged 16–24 in Tajikistan (results published) [92], women aged 18–24 in Palestine and women aged 16–24 in Bolivia [93] (results forthcoming).

## Additional files

Additional file 1 The discussion guide used in focus group discussions and interviews with the target group. (PDF 380 kb)

Additional file 2 The discussion guide used in interviews with providers. (PDF 289 kb)

Additional file 3 The matrix that displays the change objectives by crossing the determinants and performance objectives. (PDF 354 kb)

Additional file 4 The behaviour change methods included in the intervention, the basis upon which they were selected, determinants, parameters for effectiveness, and how the parameters were taken into account. (PDF 193 kb)

## Abbreviations

FGD: Focus group discussion
IBM: Integrated Behavioural Model
IM: Intervention Mapping
IPPF: International Planned Parenthood Federation
LMIC: Low and middle income countries
LSHTM: the London School of Hygiene and Tropical Medicine

## References

[1] Darroch JE, Audam S, Biddlecom A, Kopplin G, Riley T, Singh S, et al. Adding it up: the costs and benefits of investing in sexual and reproductive health 2017 (investing in contraception and maternal and newborn health fact sheet). New York: Guttmacher Institute; 2017.

[2] Hardee K, Eggleston E, Wong EL, Irwanto, Hull TH (2004). Unintended pregnancy and women's psychological well-being in Indonesia. J Biosoc Sci.

[3] Khajehpour M, Simbar M, Jannesari S, Ramezani-Tehrani F, Majd HA (2013). Health status of women with intended and unintended pregnancies. Public Health.

[4] Gipson JD, Koenig MA, Hindin MJ (2008). The effects of unintended pregnancy on infant, child, and parental health: a review of the literature. Stud Fam Plan.

[5] Najman JM, Morrison J, Williams G, Andersen M, Keeping JD (1991). The mental health of women 6 months after they give birth to an unwanted baby: a longitudinal study. Soc Sci Med.

[6] Barber JS, Axinn WG, Thornton A (1999). Unwanted childbearing, health, and mother-child relationships. J Health Soc Behav.

[7] Nakku JEM, Nakasi G, Mirembe F (2006). Postpartum major depression at six weeks in primary health care: prevalence and associated factors. Afr Health Sci.

[8] Lau Y, Keung DWF (2007). Correlates of depressive symptomatology during the second trimester of pregnancy among Hong Kong Chinese. Soc Sci Med.

[9] Orr ST, Miller CA (1997). Unintended pregnancy and the psychosocial well-being of pregnant women. Womens Health Issues.

[10] Cheng D, Schwarz EB, Douglas E, Horon I (2009). Unintended pregnancy and associated maternal preconception, prenatal and postpartum behaviors. Contraception.

[11] Eggleston E (2000). Unintended pregnancy and women’s use of prenatal care in Ecuador. Social Sci Med.

[12] Marston C, Cleland J (2003). Do unintended pregnancies carried to term lead to adverse outcomes for mother and child? An assessment in five developing countries. Popul Stud.

[13] Kost K, Landry DJ, Darroch JE (1998). Predicting maternal behaviors during pregnancy: does intention status matter?. Fam Plan Perspect.

[14] Magadi MA, Madise NJ, Rodrigues RN (2000). Frequency and timing of antenatal care in Kenya: explaining the variations between women of different communities. Social Sci Med.

[15] Shah PS, Balkhair T, Ohlsson A, Beyene J, Scott F, Frick C (2011). Intention to become pregnant and low birth weight and preterm birth: a systematic review. Matern Child Health J.

[16] Mohllajee AP, Curtis KM, Morrow B, Marchbanks PA (2007). Pregnancy intention and its relationship to birth and maternal outcomes. Obstet Gynecol.

[17] Carson C, Redshaw M, Sacker A, Kelly Y, Kurinczuk JJ, Quigley MA (2013). Effects of pregnancy planning, fertility, and assisted reproductive treatment on child behavioral problems at 5 and 7 years: evidence from the millennium cohort study. Fertil Steril.

[18] Brown SS, Eisenberg L (1995). The best intentions: unintended pregnancy and the well-being of children and families.

[19] Sedgh G, Henshaw S, Singh S, Ahman E, Shah IH (2007). Induced abortion: estimated rates and trends worldwide. Lancet (London, England).

[20] Unsafe Abortion (2011). Global and regional estimates of the incidence of unsafe abortion and associated mortality in 2008.

[21] Sedgh G, Ashford L, Hussain R (2016). Unmet need for contraception in developing countries: examining Women’s reasons for not using a method.

[22] Darroch JE, Woog V, Bankole A, Ashford L (2016). Adding it up: costs and benefits of meeting the contraceptive needs of adolescents.

[23] Tajikistan Demographic and Health Survey 2012. Dushanbe, Tajikistan, and Calverton, Maryland, USA: SA, MOH, and ICF international.: Statistical agency under the president of the Republic of Tajikistan (SA), Ministry of Health [Tajikistan], and ICF International., 2013. https://dhsprogram.com/pubs/pdf/fr279/fr279.pdf.

[24] Bertrand J. USAID Graduation from family planning assistance: implications for Latin America Tulane University School of public health and tropical Medicine, 2011.

[25] Encuesta Nacional de Demografía y Salud 2008. 2009. https://www.dhsprogram.com/pubs/pdf/FR228/FR228%5B08Feb2010%5D.pdf. Accessed 26 Apr 2018.

[26] UNFPA. Independent Country Programme Evaluation-Bolivia. 2012. http://www.unfpa.org/admin-resource/bolivia-country-programme-evaluation. Accessed 26 Apr 2018.

[27] Roudi-Fahimi F, Abdul Monem A. Unintended pregnancies in the Middle East and North Africa. In: Bureau PR, editor. 2010.

[28] Palestinian Central Bureau of Statistics. Palestinian Family Health Survey 2006: Final Report. Ramallah, Palestine: 2007. http://www.pcbs.gov.ps/pcbs-metadata-en-v4.3/index.php/catalog/116.

[29] Palestinian Central Bureau of Statistics. Palestinian Multiple Indicator Cluster Survey 2014: Final Report. Ramallah, Palestine: 2015. http://www.pcbs.gov.ps/Downloads/book2099.pdf.

[30] Courbage Y, Abu Hamad B, Zagha A. Palestine 2030- demographic change: opportunities for development. State of Palestine: United Nations Population Fund Palestine; 2016.

[31] Cleland J, Ali MM (2004). Reproductive consequences of contraceptive failure in 19 developing countries. Obstet Gynecol.

[32] Trussell J, Hatcher R, Trussell J, Nelson A, Cates W, Kowal D, Policar M (2011). Contraceptive efficacy. Contraceptive technology: twentieth revised edition.

[33] Trussell J. Contraceptive efficacy: global library of women's medicine; 2014 [26 September 2017]. Available from: http://www.glowm.com/section_view/heading/Contraceptive%20Efficacy/item/374.

[34] Bradley SEK, Croft TN, Fishel JD, Westoff CF. Revising unmet need for family planning. Calverton, Maryland, USA: ICF International, 2012.

[35] Palestinian Central Bureau of Statistics. Final Report of the Palestinian Family Survey 2010. Ramallah, Palestine: 2013. http://www.pcbs.gov.ps/Downloads/book1941.pdf.

[36] Hammoudeh W (2014). Addressing family planning delivery gaps in the Palestinian territory.

[37] Hall KS, Westhoff CL, Castano PM (2013). The impact of an educational text message intervention on young urban women’s knowledge of oral contraception. Contraception.

[38] Mobile 4 Reproductive Health- Evidence 2013. Available from: http://m4rh.fhi360.org/?page_id=12.

[39] Castaño PM, Bynum JY, Andres R, Lara M, Westhoff C (2012). Effect of daily text messages on oral contraceptive continuation: a randomized controlled trial. Obstet Gynecol.

[40] Berenson AB, Rahman M (2012). A randomized controlled study of two educational interventions on adherence with oral contraceptives and condoms. Contraception.

[41] Trent M, Thompson C, Tomaszewski K (2015). Text messaging support for urban adolescents and young adults using injectable contraception: outcomes of the DepoText pilot trial. J Adolesc Health.

[42] Smith C, Ngo TD, Gold J, Edwards P, Vannak U, Sokhey L, et al. Effect of a mobile phone-based intervention on post-abortion contraception: a randomized controlled trial in Cambodia. Bull World Health Organ. 2015; 10.2471/BLT.15.160267 PMC4669734 26668436

[43] Halpern V, Lopez LM, Grimes DA, Stockton LL, Gallo MF (2013). Strategies to improve adherence and acceptability of hormonal methods of contraception. Cochrane Database Syst Rev.

[44] Lopez LM, Grey TW, Chen M, Tolley EE, Stockton LL (2016). Theory-based interventions for contraception. Cochrane Database Syst Rev.

[45] Smith C, Gold J, Ngo TD, Sumpter C, Free C. Mobile phone-based interventions for improving contraception use. Cochrane Database Syst Rev. 2015;26(6):CD011159. 10.1002/14651858.CD011159.pub2 PMC6485989 26115146

[46] Higgins JP, Altman DG, Gotzsche PC, Juni P, Moher D, Oxman AD (2011). The Cochrane Collaboration's tool for assessing risk of bias in randomised trials. BMJ.

[47] Bartholomew Eldredge LK, Markham C, Ruiter R, Fernandez M, Kok G, Parcel G (2016). Planning health promotion programs: an intervention mapping approach.

[48] Kok G, Gottlieb N, Peters G, Mullen P, Parcel G, Ruiter R (2016). A taxonomy of behavior change methods; an intervention mapping approach. Health Psychol Rev.

[49] McCarthy O, Carswell K, Murray E, Free C, Stevenson F, Bailey JV (2012). What young people want from a sexual health website: design and development of Sexunzipped. J Med Internet Res.

[50] Free C, McCarthy O, French R, Wellings K, Michie S, Roberts I (2016). Can text messages increase safer sex behaviours in young people? Intervention development and pilot randomised controlled trial. Health Technol Assess.

[51] United Nations Department of Economic and Social Affairs. Definition of Youth [26 September 2017]. Available from: http://www.un.org/esa/socdev/documents/youth/fact-sheets/youth-definition.pdf.

[52] Deliver + Enable Toolkit: Scaling-up comprehensive sexuality education (cse) London: The International Planned Parenthood Federation; 2016. Available from: https://www.ippf.org/resource/deliverenable-toolkit-scaling-comprehensive-sexuality-education-cse.

[53] Abraham C, Michie S (2008). A taxonomy of behavior change techniques used in interventions. Health Psychol.

[54] Lopez LM, Tolley EE, Grimes DA, Chen M, Stockton LL (2013). Theory-based interventions for contraception. Cochrane Database Syst Rev.

[55] Gottschalk LB, Ortayli N (2014). Interventions to improve adolescents’ contraceptive behaviors in low- and middle-income countries: a review of the evidence base. Contraception.

[56] Montaño D, Kasprzyk D, Glanz K, Rimer BK, Viswanath K (2015). Theory of reasoned action, theory of planned behavior, and the integrated behavioral model. Health behaviour: theory, research and practice.

[57] Fisher JD, Fisher WA (1992). Changing AIDS-risk behavior. Psychol Bull.

[58] Bongaarts J, Bruce J (1995). The causes of unmet need for contraception and the social content of services. Stud Fam Plan.

[59] Glinski A, Sexton M, Petroni S. Understanding the adolescent family planning evidence base. Washington, D.C.: International Center for Research on Women; 2014.

[60] Wells E (2015). Countering myths and misperceptions about contraceptives.

[61] Campbell M, Sahin-Hodoglugil NN, Potts M (2006). Barriers to fertility regulation: a review of the literature. Stud Fam Plan.

[62] Chandra-Mouli V, McCarraher DR, Phillips SJ, Williamson NE, Hainsworth G (2014). Contraception for adolescents in low and middle income countries: needs, barriers, and access. Reprod Health.

[63] Cleland J, Bernstein S, Ezeh A, Faundes A, Glasier A, Innis J (2006). Family planning: the unfinished agenda. Lancet.

[64] Casterline JB, Sathar ZA, ul Haque M (2001). Obstacles to contraceptive use in Pakistan: a study in Punjab. Stud Fam Plan.

[65] Sedgh G, Hussain R (2014). Reasons for contraceptive nonuse among women having unmet need for contraception in developing countries. Stud Fam Plan.

[66] Williamson LM, Parkes A, Wight D, Petticrew M, Hart GJ (2009). Limits to modern contraceptive use among young women in developing countries: a systematic review of qualitative research. Reprod Health.

[67] Wulifan JK, Brenner S, Jahn A, De Allegri M (2016). A scoping review on determinants of unmet need for family planning among women of reproductive age in low and middle income countries. BMC Womens Health.

[68] Sedgh G, Hussain R, Bankole A, Singh S. Women with an unmet need for contraception in developing countries and their reasons for not using a method. New York: Guttmacher Institute, 2007 37.

[69] Darroch JE, Sedgh G, Ball H (2011). Contraceptive technologies: responding to Women’s needs.

[70] Casterline JB, Perez AE, Biddlecom AE (1997). Factors underlying unmet need for family planning in the Philippines. Stud Fam Plan.

[71] Stash S (1999). Explanations of unmet need for contraception in Chitwan, Nepal. Stud Fam Plan.

[72] Ochako R, Mbondo M, Aloo S, Kaimenyi S, Thompson R, Temmerman M (2015). Barriers to modern contraceptive methods uptake among young women in Kenya: a qualitative study. BMC Public Health.

[73] Fishbein M, Ajzen I (1975). Belief, Attitude, Intention, And behavior: an introduction to theory and research. Reading.

[74] Ajzen I (1988). Attitudes, Personality and behavior.

[75] Bandura A (2006). Toward a psychology of human agency. Perspect Psychol Sci.

[76] Bandura A (1986). Social foundations of thought and action: a social cognitive theory.

[77] Kelder S, Hoelscher D, Perry CL, Glanz K, Rimer BK, Viswanath K (2015). How individuals, environments and health behaviors interact: social cognitive theory. Health behavior: theory, research and practice.

[78] Ajzen I (1991). The theory of planned behavior. Organ Behav Hum Decis Process.

[79] Coyle K, Basen-Engquist K, Kirby D, Parcel G, Banspach S, Collins J, et al. Safer choices: reducing teen pregnancy, HIV, and STDs. Public Health Rep (Washington, D C : 1974) 2001;116 Suppl 1:82–93. 10.1093/phr/116.S1.82 PMC1913682 11889277

[80] Sieving RE, McRee A-L, McMorris BJ, Beckman KJ, Pettingell SL, Bearinger LH (2013). Prime time: sexual health outcomes at 24 months for a clinic-linked intervention to prevent pregnancy risk behaviors. JAMA Pediatr.

[81] Schinke SP, Blythe BJ, Gilchrist LD.. Cognitive-behavioral Prevention of adolescent pregnancy. J Couns Psychol 1981;28(5):451–454.

[82] Canto De Cetina T, Canto P, Ordonez Luna M (2001). Effect of counseling to improve compliance in Mexican women receiving depot-medroxyprogesterone acetate. Contraception.

[83] Ingersoll KS, Ceperich SD, Nettleman MD, Karanda K, Brocksen S, Johnson BA (2005). Reducing alcohol-exposed pregnancy risk in college women: initial outcomes of a clinical trial of a motivational intervention. J Subst Abus Treat.

[84] Floyd RL, Sobell M, Velasquez MM, Ingersoll K, Nettleman M, Sobell L (2007). Preventing alcohol-exposed pregnancies: a randomized controlled trial. Am J Prev Med.

[85] Peters GA. Practical guide to effective behavior change: how to identify what to change in the first place. The European health. Psychologist. 2014;16(5)

[86] Jarallah Y. Marriage patterns in Palestine. Population Reference Bureau, 2008.

[87] Rashad H, Osman M, Roudi-Fahimi F (2005). Marriage in the Arab world.

[88] Hou MY, Hurwitz S, Kavanagh E, Fortin J, Goldberg AB (2010). Using daily text-message reminders to improve adherence with oral contraceptives: a randomized controlled trial. Obstet Gynecol.

[89] L'engle K, Vahdat H, Ndakidemi E, Lasway C, Zan T (2013). Evaluating feasibility, reach and potential impact of a text message family planning information service in Tanzania. Contraception.

[90] Vahdat HL, L'Engle KL, Plourde KF, Magaria L, Olawo A (2013). There are some questions you may not ask in a clinic: providing contraception information to young people in Kenya using SMS. Int J Gynaecol Obstet.

[91] ICT Facts and Figures 2016. International Telecommunication Union: http://www.itu.int/en/ITU-D/Statistics/Documents/facts/ICTFactsFigures2016.pdf, 2016.

[92] McCarthy O, Ahamed I, Kulaeva F, Tokhirov R, Saibov S, Vandewiele M (2018). A randomized controlled trial of an intervention delivered by mobile phone app instant messaging to increase the acceptability of effective contraception among young people in Tajikistan. Reprod Health.

[93] McCarthy OL, Osorio Calderon V, Makleff S, Huaynoca S, Leurent B, Edwards P (2017). An intervention delivered by app instant messaging to increase acceptability and use of effective contraception among young women in Bolivia: protocol of a randomized controlled trial. JMIR Res Protoc.

