# Supplementary material for: Development of an intervention delivered by mobile phone aimed at decreasing unintended pregnancy among young people in three lower middle income countries
Source: BMC Public Health. 2018 May 2;18:576. doi: 10.1186/s12889-018-5477-7 (PMC5930955; doi:10.1186/s12889-018-5477-7)
Supplement: Supplementary file 1 — The discussion guide used in focus group discussions and interviews with the target group. (PDF 380 kb) [file 12889_2018_5477_MOESM1_ESM.pdf]

Additional File 1. Target group discussion guide

1. **Introduction**
   - We would like to hear about young people's experiences and thoughts about contraception so that we can best help them
   - We also would like to know what young people about receiving contraceptive support by mobile phone
   - Don't have to take part if you don't want to
   - There are no right or wrong answers
   - I am here to facilitate the discussion, I'm not an expert on the issues
   - We are interested in a range of views, so it's ok to disagree with each other
   - Check again that they are ok with audio recording, explain confidentiality and anonymity
   - It will last up to 90 mins (up to 60 mins for interview)
   - You can leave when you want without having to give a reason
   - You don't have to talk about anything you don't want to talk about

2. **Informed consent**
   - Give them two copies of the consent, one is for them and one for you
   - Ask them to initial each box, print, sign and date
   - They don't have to add their number if they don't want to

3. **Demographic questionnaire**
   - This information is anonymous, your name won't be on it
   - It's so we can describe the kinds of people we've talked to

Rules (for focus group):

- Please respect each other's confidentiality by not sharing anything with anyone outside the group that might identify people in the group
- Respect each other's opinions
- Don't interrupt
- Please one person speak at a time so that we can understand the recording
- Can take phone calls (please leave the room for the conversation)
- Any questions before I ask you to provide consent and fill out a questionnaire?

4. **Game**
   - Ideas: each person says their name and then one thing about themselves and then people guess if it's true or false, each person says their name and something about themselves and each person has to remember wha tht epeople before them have said, another game that you may know of

5. **Technology & information**

Aim: to understand patterns of technology use and what tools they use to access information (general and contraception)

- Where do young people get their information from? [If you wanted to find out about something, where would you go (e.g. websites, apps, friends, books, friends, parents]?
- Do you own a phone? [How many? What type? How often do you replace your main phone? (if they don't own one) Do you use someone else's phone?]
- Do you share your phone ever? [e.g. with friends or family. If yes, how do you keep things on your phone private?]
- What do you use your phone for? [if apps, which ones?]
- Do you have access to the internet? [where, how and when?]
- When do you get information from your phone? [are there times that you can't get info from your phone?]
- **[MUST ASK] What would young people think about receiving contraceptive information about on their mobile phone?**
- How helpful is contraception support on your phone if someone is not having sex?

- How would you like to receive contraceptive support on your phone? (e.g. text, app, voice message, instant message)

To change and add for the next group/interview:

6. Knowledge

Aim: to find out how much they know about the range of methods so we can identify any gaps in knowledge

- What kinds of contraception do young people know about? [show images of different methods, ask about traditional methods]
- What do you know about them (the methods that they named)? [How do you use (methods)?]
- How do young people find out about contraception? [Where would you go to find out more? How do your friends learn about it?]
- Would young people like more information about contraception? [how would you like to receive this information?]
- (F) If you have gone to a reproductive health centre/youth friendly centre, what have the providers told you about the different types of contraception?
- How easy do you think is it for a woman to get pregnant if they are not using any contraception?
- How easy do you think it is for a woman to get pregnant if they are just using condoms?

To change and add for the next group/interview:

7. Attitudes

Aim: to identify misconceptions, biases, perceptions and stigma surrounding contraception.

- What do people in Bolvia think about people who use contraception? [Why do you think this? Married/unmarried]
- What do young people think about contraception? [How many of your friends use it? What kinds do they use?]
- Show images of different methods and ask what they think about each.
- What concerns (if any) do you have about these different methods? [Do you think any of them are harmful? (if yes- which ones? Why do you think this?)]
- Who should take responsibility for contraception? [you, your partner or both?]
- What do you think about unmarried young people having sex? [Why? Different for females & males?]
- How does contraception make you feel/would contraception make you feel about sex? [more or less pleasurable? Why?]

To change and add for the next group/interview:

8. Barriers

Aim: to explore things that may prevent them from using contraception.

- How common it is for young people to use contraception? [ones have you/they used? What was your/their experience using the different methods? If not common, ask why not]
- What kinds of problems (if any) do you or other people have with using contraception?
- How easy is it for young people to get to the clinic/mobile service? [rural/urban]
- How do you think providers feel about an unmarried person asking for contraception?
- A young woman in her teens just got married. How acceptable is it for her to wait to get pregnant?
- What role does religion play in how acceptable contraception is? [does this influence how you feel about it?]
- What do you think about women making decisions on their own about contraception? [how easy/hard is it for them? Why do you think this?]
- Does the cost of contraception prevent people from using it?
- (F) If your partner didn't approve of contraception, would you use it anyway? [how important is it that your partner approves?]

To change and add for the next group/interview:

9. Communication

Aim: to understand how confident they are talking to partners and others about their reproductive needs and preferences.

- How confident are young people with talking to their partner about contraception? [what would help them feel more confident?]
- How do young people feel about talking to staff at the clinic about contraception? [F- are young women comfortable talking to them on their own? Why/why not?]
- How confident are young people with talking to their friends about contraception?
- How confident are young people with talking to their parents about contraception?
- If a young woman told her partner that she wanted to use contraception but her (male) partner said he didn't want to, what would happen?
- Imagine a friend of yours is having sex and doesn't want a pregnancy at this time. What would you say to her/him?

To change and add for the next group/interview:

10. Trying new methods

Aim: to get a sense for how open they are to trying new methods and how confident they feel about trying them.

- (F) If a young person wanted to try a new method, how confident would they be about trying it? [how easy/difficult would it be? What would prevent you from trying it?]
- (F) What would help young people feel more confident trying new methods? [show images if they don't respond]
- What would need to change to make people feel more confident in using contraception?
- What kinds of contraception do you think young people would be interested in trying?

To change and add for the next group/interview:

11. Intervention content & privacy

Aim: to find out how the intervention can be supportive & easy to understand.

- What kinds of messages should we send? [e.g. educational, stories and quotes from other people about using contraception, ideas about how to talk about contraception with your partner, images, etc.]
- What kinds of messages should we not send?
- What concerns would young people have about receiving messages like these on your mobile phone?
- How many messages should we send each day (if it was delivered by text or instant message)?

To change and add for the next group/interview:

12. Wrap up

- Thinking about all that we talked about today, what do you feel is the most important? [why?]
- What we've talked about today will help us provide the best contraceptive support by mobile phone
- LAST QUESTION- if you were part of a study, would you come to the service so that the researchers could check your contraceptive use?
- Any questions or comments?
- Thank you! (if they want any more information, show our contact details again)
