# Supplementary material for: Development of an intervention delivered by mobile phone aimed at decreasing unintended pregnancy among young people in three lower middle income countries
Source: BMC Public Health. 2018 May 2;18:576. doi: 10.1186/s12889-018-5477-7 (PMC5930955; doi:10.1186/s12889-018-5477-7)
Supplement: Supplementary file 2 — The discussion guide used in interviews with providers. (PDF 289 kb) [file 12889_2018_5477_MOESM2_ESM.pdf]

Additional File 2. Provider discussion guide

1. **Introduction**
   - We are asking people to help us develop content for a mobile phone contraceptive intervention.
   - We'd like to hear about your experiences and thoughts about the service users' contraceptive use.
   - We also would like to know what you think about receiving contraceptive support by mobile phone.
   - Check again that they are ok with audio recording, explain confidentiality and anonymity
   - It will last up to 60 mins
   - Can take phone calls if you need to
   - You can leave when you want without having to give a reason
   - You don't have to talk about anything you don't want to talk about
   - Any questions before we start?

2. **Informed consent**
   - Give them two copies of the consent, one is for them and one for you
   - Ask them to initial each box, print, sign and date
   - They don't have to add their number if they don't want to

3. **Demographic questionnaire**
   - This information is anonymous, your name won't be on it
   - It's so we can describe the different people we've talked to

4. **Knowledge**

Aim: to find out their perceptions regarding users' knowledge about the range of methods

- What kinds of contraception do you think that the service users know about?
- What don't they know about? [why do you think they don't know about them?]
- Do you think service users want more information about contraception? [if yes- how would they like to receive this information? Why do you think this? If no, why not?]
- Where do you think users get information in general? [e.g. online web forums, friends, books]
- Where do they get information about contraception?
- What do service users think about contraception in general?
- What do users think about (name each method)? [why do they have these views?]
- What do users say about different methods? [have they expressed any concerns, e.g. side effects?]

To change and add for the next interview:

5. **Attitudes**

Aim: to find out their perceptions regarding user's attitudes towards contraception

- What methods do you think are best for young people? [why? Married/unmarried]
- What methods do you think are not appropriate for young people? [why? Married/unmarried]
- What would you recommend for a young unmarried women who wants contraception? [why?]
- What is the best method for a young woman in a relationship who has never been pregnant but wants a family someday? [why?]
- Are there any methods that you think are unsafe? Why do you think this? [Is this your experience or have you heard this from other people?]
- How safe are IUDs? [Why do you think this?]

To change and add for the next interview:

6. **Barriers**

Aim: to find out what they think prevents users from using contraception.

- What prevents service users from using effective contraception?
- How acceptable is contraception in Bolivia

- How does (religion, women's status in society, stigma, social-disapproval in general) influence contraception use? [Do you think this can change and if so, how?]
- What kinds of problems do people have with using contraception? [financial, supply, access, language]
- What influence do parents/friends have on users' choice?
- How important is it partner approval in people's choice?
- What would need to change to make people feel more confident in using effective contraception?

To change and add for the next interview:

7. Communication

Aim: to find out what they think about the frequency and quality of users' communication about contraception with partners & providers

- What kinds of things do you discuss with a person who is looking for contraception? [married & not married]
- What kinds or methods do you suggest for them? [Ask why for each and ask why they do not recommend others. How do they respond?]
- How comfortable to users seem when they talk to you about contraception?

To change and add for the next interview:

8. Trying new methods

Aim: to hear providers' opinions on how open people are to trying new methods

- What would help young people feel more confident trying new methods?

To change and add for the next interview:

9. Technology

Aim: to hear their views on user acceptability of a mobile phone intervention.

- Do you think young people would like to receive information by mobile phone? [why or why not? If yes, what kind of information?]
- How would you see a mobile phone intervention fitting into the services that you provide?

To change and add for the next interview:

10. Wrap up

- Thinking about all that we talked about today, what do you feel is the most important? [why?]
- What we've talked about today will help us provide the best contraceptive support by mobile phone
- Any questions or comments?
- Thank you! (if they want any more information, show our contact details again)
