# Supplementary material for: Development of an intervention delivered by mobile phone aimed at decreasing unintended pregnancy among young people in three lower middle income countries
Source: BMC Public Health. 2018 May 2;18:576. doi: 10.1186/s12889-018-5477-7 (PMC5930955; doi:10.1186/s12889-018-5477-7)
Supplement: Supplementary file 3 — The matrix that displays the change objectives by crossing the determinants and performance objectives. (PDF 354 kb) [file 12889_2018_5477_MOESM3_ESM.pdf]

Additional file 3. Matrix of change objectives

	Determinants			
**Performance objectives**				
	**Knowledge**	**Attitude**	**Intention**	**Personal agency**
Young people…	*Behavioral outcome 1: Use effective contraception*			
po1.1 Choose a method	k1.1.1 Name the effective methods<br><br>k1.1.2 Describe how the effective methods work<br><br>k1.1.3 List the risks & benefits of the range of effective methods	a1.1.1 Express positive attitudes towards the effective methods<br><br>a1.1.2 Recognise that hormonal methods are not less healthy than non-hormonal methods<br><br>a1.1.3 Differentiate between real potential side-effects and misconceptions<br><br>a1.1.4 Recognise that an experience of side-effects in one method may not occur in another method	i1.1.1 Assess options<br><br>i1.1.2 Express intention to choose effective contraception	pa1.1.0 Express personal agency in choosing an effective method despite fears of being judged by society (married or not married)
po1.2 Acquire the method	k1.2.0 List services that provide effective contraception	a1.2.1/a2.1.1<br>Question whether not attending a service because of fear of being judged by providers is worth the risk of unintended pregnancy<br><br>a1.2.2 Question whether not using effective contraception because of fear of being judged by society (whether married or unmarried) is worth the risk of unintended pregnancy<br><br>a1.2.3 Recognise that it is better to withhold assumptions about the affordability of contraception until the price has been confirmed by the service	i1.2.0 Express intention to acquire effective contraception	

	Determinants			
**Performance objectives**				
	**Knowledge**	**Attitude**	**Intention**	**Personal agency**
po1.3 Use the method correctly	k1.3.0 Describe how methods are used		i1.3.0 Express intention to use effective contraception	pa1.3.0 Express personal agency in using methods correctly
	Behavioral outcome 2: Access reproductive health services			
po2.1 Locate a service	k1.2.0/2.1.0 List services that provide effective contraception	a2.1.1/a1.2.1<br>Question whether not attending a service because of fear of being judge by providers is worth the risk of unintended pregnancy<br><br>a2.1.2 Understand that it is fundamental to providers' job to maintain confidentiality<br><br>(B)a2.1.3 Debate cultural norm of not accessing services until something is wrong	i2.1.0 Express intention to locate a service	
po2.2 Travel to the service			i2.2.0 Plan travel to the service<br><br>i2.2.1 Recognise that it is possible for young people (married or not married) to travel to services while preserving privacy	pa2.2.0 Express personal agency in travelling to the service
po2.3 Communicate effectively with providers				pa2.3.0 Express personal agency in communicating with providers
	Behavioral outcome 3: Communicate with partners about contraception before sex			

	Determinants			
**Performance objectives**				
	**Knowledge**	**Attitude**	**Intention**	**Personal agency**
po3.1 Initiate conversation with partner		a3.1.1 Question whether refraining from initiating a conversation about contraception with a partner is worth the risk of unintended pregnancy<br><br>(B)a3.1.3 Assess whether having sex without using effective contraception is really a “proof of love”	i3.1.0 Express intention to initiate conversation about effective contraception with partners	pa3.1.0 Express personal agency in initiating a conversation about effective contraception with partners
po3.2 State own fertility preferences			i3.2.0 Express intention to clearly state own fertility preferences with partner	pa3.2.0 Express personal agency in clearly stating own fertility preferences to partner
po3.3 Listen to partner’s fertility preferences			i3.3.0 Express intention to listen to partner’s fertility preferences	pa3.3.0 Express personal agency in listening to partners talk about their fertility preferences

(B) Bolivia only
