# Supplementary material for: Development of an intervention delivered by mobile phone aimed at decreasing unintended pregnancy among young people in three lower middle income countries
Source: BMC Public Health. 2018 May 2;18:576. doi: 10.1186/s12889-018-5477-7 (PMC5930955; doi:10.1186/s12889-018-5477-7)
Supplement: Supplementary file 4 — The behaviour change methods included in the intervention, the basis upon which they were selected, determinants, parameters for effectiveness, and how the parameters were taken into account. (PDF 193 kb) [file 12889_2018_5477_MOESM4_ESM.pdf]

Additional file 4. Behaviour change methods

Method	Definition	The basis upon which the method was selected	Determinant	Parameters	How the parameters were taken into account
Belief selection	Using messages designed to strengthen positive beliefs, weaken negative beliefs and introduce new beliefs	Theory of planned behaviour, Reasoned action approach	Knowledge, Attitude	Requires investigation of the current attitudinal, normative and efficacy beliefs of the individual before choosing the beliefs on which to intervene	Beliefs targeted were identified in the needs assessment
Facilitation	Creating an environment that makes the action easier or reduces barriers to action	Social cognitive theory, suitability for delivery by text message	Intention, Personal agency	Requires real changes in the environment instead of perceptions of the environment; requires the identification of barriers and facilitators and the power for making the appropriate changes.	Cannot produce real changes in the environment but provides advice on how they can change their environment to remove barriers
Anticipated regret	Stimulating individuals to focus on their feelings after unintended risky behaviour, before any losses actually materialise	Theory of planned behaviour, Reasoned action approach	Knowledge, Attitude, Intention, Personal agency	Stimulation of imagery; assumes a positive intention to avoid the risky behaviour	Cannot be certain that the individual intends to avoid the behaviour. However, the intervention aims to influence intention.
Guided practice	Prompting individuals to rehearse and repeat the behaviour various times, discuss the experience and provide feedback	Social cognitive theory	Intention, Personal agency	Subskill demonstration, instruction and enactment with individual feedback; requires supervision by an experienced person	Cannot provide feedback or supervision. However, real quotes from the target group will help participants compare themselves

Method	Definition	The basis upon which the method was selected	Determinant	Parameters	How the parameters were taken into account
					with an example of a conversation that went well.
Verbal persuasion	Using messages that suggest that the participant possesses certain capabilities	Social cognitive theory	Intention, Personal agency	Credible source	Individuals will know that the messages are coming from the organisation and were written the organisation, the University of London and young people
Tailoring	Matching the intervention or components to previously measured characteristics of the participant	Needs assessment	Knowledge	Tailoring variable or factors related to behaviour change (such as stage) or to relevance (such as culture)	The intervention is based on the needs assessment and is tailored according to marital status and gender
Cultural similarity	Using characteristics of the target group in source, message and channel	Needs assessment	Attitude	Using surface characteristics of the target group enhances receptivity. Socio-cultural characteristics leads to a more positive reception of the message.	Quotes from people in the target group are used
Arguments	Using a set of one or more meaningful premises and a conclusion	Suitability for delivery by text message	Attitudes, Intention, Personal agency	Arguments need to be new	Cannot be certain that all arguments were new to individuals but they might be

Method	Definition	The basis upon which the method was selected	Determinant	Parameters	How the parameters were taken into account
Shifting perspective	Encouraging taking the perspective of the other	Needs assessment	Personal agency	Initiation from the perspective of the individual; needs imaginary competence	Cannot be certain that that the individual has imaginary competence. The needs assessment revealed lack of confidence in communicating with providers so this method prompts individuals to see the situation from the providers’ perspective.
Goal setting	Prompting planning what the person will do, including a definition of goal-directed behaviours that results in the target behaviour	Suitability for delivery by text message	Intention	Commitment to the goal; goals that are difficult but available within the individual's skill level	Cannot know if individuals are committed to the goal or if it is within their skill level. However, it might be.
Accurate information provision		Information-Motivation-Behavioural Skills Model; not a method, and generally not an important predictor of behaviour, but in contexts where knowledge is low, providing information may influence behaviour	Knowledge		
